# Establishing Background Pathologic Changes of Valve Replacement Surgery in Sheep

**DOI:** 10.1007/s13239-021-00563-6

**Published:** 2021-07-14

**Authors:** Jill T. Schappa Faustich, John P. Carney, Matthew T. Lahti, Benjamin L. Zhang, Richard W. Bianco

**Affiliations:** Experimental Surgical Services Laboratory, Department of Surgery, University of Minnesota, Minneapolis, MN 55455 USA

**Keywords:** Animal model, Valve replacement surgery, Cardiopulmonary bypass, Renal infarcts, Preclinical, Sheep

## Abstract

**Purpose:**

Sheep are the standard preclinical model for assessing safety of novel replacement heart valves, yet the anatomic and pathologic effects of invasive surgery, including those involving cardiopulmonary bypass (CPB), are unknown. Thus, we aimed to determine the gross, hematologic and biochemical effects of sham mitral and aortic replacement valve procedures in sheep to establish a useful control for evaluation of novel replacement valves.

**Methods:**

Six control sheep were examined without any surgical intervention. Six sham mitral valve replacements (MVR) and six sham aortic valve replacements (AVR) were performed on 12 sheep. Complete blood counts and serum biochemistry were performed throughout the study. Sheep were sacrificed with a necropsy performed at 90 days.

**Results:**

Renal infarcts (RIs) were the most frequently observed lesion, averaging 4.7 in control sheep, 2.5 with MVR and 5.8 with AVR. The number of infarcts strongly correlated with total estimated area of infarcted kidney (*r* = .84, *p* < .01). Additional cardiac interventions were significantly correlated with increased numbers of RIs (*r* = .85, *p* < .01). There was no correlation between number of RIs and time on CPB, or between AVR and MVR procedures.

**Conclusion:**

The sheep model for AVR and MVR requires invasive surgery and CPB, which are associated with background anatomic and pathologic changes, especially in cases with additional surgical cardiac interventions. These findings serve as a critical control for future evaluation and development of novel replacement valves in order to distinguish device-related safety issues from expected outcomes of the surgical procedure and normal background changes in sheep.

**Supplementary Information:**

The online version contains supplementary material available at 10.1007/s13239-021-00563-6.

## Introduction

Surgical implantation of functional valve substitutes have been a long-standing treatment for appropriately selected patients with severe heart valve disease, improving life expectancy and quality of life.^3^,^18^ As a class III medical device, functional heart valve substitutes require intensive testing as mandated by the Food and Drug Administration and International Organization for Standardization (ISO).^4^ Part of the process of pre-clinical testing of these valve substitutes involves the use of large animal models for in-*vivo* safety evaluation. Sheep are a well-established animal model for this purpose and are a commonly used large animal model in testing cardiac valves.^9^ To date, very few studies have addressed the baseline pathophysiologic effects of invasive surgery, including cardiopulmonary bypass (CPB) and cardiotomy in the sheep model. Establishing baseline effects of surgery can greatly aid in differentiating true device-related safety concerns from expected background changes associated with valve implantation in the preclinical evaluation of novel replacement valves.

We aimed to determine the gross, histopathological, hematologic and biochemical effects of a sham mitral valve replacement (MVR) and aortic valve replacement (AVR) in sheep. In our extensive experience with pre-clinical testing of various device implants in sheep, we have observed frequent pathologic consequences thought to be surgically-related and not device-specific. The most notable of these is the presence of small numbers of renal infarcts. We hypothesized that valve-replacement surgery would result in common pathophysiologic alterations that could be used to establish model-specific parameters for distinguishing between procedural-related and device-related injuries in sheep.

## Materials and Methods

The animals used in this work received care in compliance with the protocols approved by the laboratory’s Institutional Animal Care and Use Committee in accordance with the guidelines for humane care.

A total of 19 ovine were used for the study. Six sheep were examined without surgical intervention to serve as controls. Seven sheep were used for the MVR sham surgery, and six sheep were used for the AVR sham surgery. One animal in the MVR group was not included in the results as this animal was euthanized during the surgical procedure due to an operative complication.

After general anesthesia and preoperative preparation, including insertion of a catheter into the jugular vein for periprocedural fluid and medication administration, a left thoracotomy was performed in the 3rd intercostal space for the aortic valve model and 4th space for the mitral valve model. The left internal mammary artery was exposed and divided for the MVR procedure to prevent arterial dissection during thoracic retraction. For the AVR procedure, the proximal segment of the mammary artery was cannulated for invasive hemodynamic monitoring with the distal segment ligated and the artery divided. Hemodynamic monitoring with a pressure line was implemented in aortic surgery to verify perfusion to the head vessels, as the ascending aorta is cross clamped for exposure of the native aortic annulus. Since aortic clamping and exposure is not necessary for mitral surgery an arterial line is not required in this procedure. The pericardium was then opened. Anticoagulation was achieved using 250 IU/kg of heparin with the activated clotting time (ACT) maintained above 300 s. Protamine was administered for reversal at the end of the procedure. Arterial cannulation was performed via the descending aorta with the cannula directed proximally. Venous cannulation was through the right atrial appendage. CPB was initiated and when adequate return to the venous reservoir was established, the animal was cooled to approximately 28 °C a standard practice common in heart valve replacement surgeries in large animals.^1^,^6^,^10^ A small stab incision was made through the center of a previously placed purse string in the left ventricular apex and an appropriately sized venous cannula was positioned within the left ventricle. Venting was established immediately at low flow.

For the MVR model, the heart was arrested using a pulse train stimulator. A left atriotomy was performed to expose the native mitral valve, which was atraumatically manipulated to simulate resection of the native mitral leaflets and placement of annular stitches. The annulus was manipulated in a way that mimicked parachuting a prosthetic valve into the annulus and securing it in place.

For the AVR model, the aorta was cross clamped proximal to the junction of the brachiocephalic trunk, a cardioplegia catheter was inserted into the aorta proximally and cold cardioplegia solution was administered until cardiac arrest occurred. A partial transverse aortotomy was performed. The native aortic valve was atraumatically manipulated to simulate resection of the native aortic leaflets and placement of annular stitches. The annulus was manipulated in a way that mimicked parachuting a prosthetic valve into the annulus and securing it in place.

Serial clinical pathology was collected at intake (1–6 days prior to surgery), on the day of surgery (pre-CPB), post-CPB (after protamine, while closing), 7 (± 1) days, monthly, and prior to sacrifice and term procedures. Intake blood samples were performed to exclude pre-existing underlying disease and assess overall health for inclusion in the study. Blood collection performed on non-anesthetized test and control group animals was performed with the animals manually restrained and a sample obtained by venipuncture to the jugular vein with a 19-gauge needle and syringe. Samples collected prior to and following CPB in anesthetized test animals were collected from the catheter inserted preoperatively for fluid and medication administration. Blood samples were processed for a complete blood count (CBC) and serum biochemistry. Anticoagulation was maintained in both groups with subcutaneous heparin (1000–2000 IU twice daily) for 2 days post-operatively. A complete necropsy was performed evaluating all internal organ systems at 90 days post-operatively. The 90 day study endpoint was chosen as it is the minimum required chronic animal study evaluation period recommended by the International Organization for Standardization (ISO) 5840-2 guidance, and has been previously published in the literature.^10^,^12^ Samples for histopathology were collected, stored in 10% neutral buffered formalin, and were routinely processed and stained with hematoxylin and eosin. Samples from both kidneys, brain, spleen, liver, both lungs, left ventricle, right ventricle, interventricular septum, and left atrium with adjacent coronary artery were evaluated by a board-certified veterinary pathologist.

At the time of necropsy, the length and width of each kidney was measured, as well as the length and width of each lesion grossly appearing consistent with a renal infarct. The height of the kidney was not recorded, thus total surface area of the kidney could not be calculated. In order to generate an estimate of the percent area of infarcted kidney, we calculated the area of the kidney and lesions as a two-dimensional model. This calculation was meant to broadly analyze whether the total number of lesions correlated with the surface of the kidney affected (in comparison to a small number of infarcts affecting a large area of the kidney). For our calculation, we summed the total surface dimensions of the infarcts (in mm^2^) and divided this area by the two-dimensional area of the kidney (in mm^2^). In this study, we use the terminology “kidney area” for simplicity, recognizing the limitations of this method of calculation.

Blood samples from the day of surgery (pre-CBP) were used as baseline controls as these values were a more accurate baseline of comparison when evaluating changes post-CBP. Intake blood samples were collected on average 3.5 days (range 1–6 days) prior to surgery and were not performed in one animal. Thus, intake samples were deemed less relevant as a baseline control as compared to pre-bypass samples. Any differences between day of surgery blood values and intake samples were minimal and not clinically relevant. Paired t-tests were performed on bloodwork post-op day 7 or 14, day 30, day 60, and day 90 in the MVR group and the AVR group as well as with both groups combined. Pearson correlation was used to examine the relationship between the number of renal infarcts (RI) and area of infarcted kidney, total time on CPB, pre- and post-CPB blood pressure, MVR and AVR procedures, and uneventful vs interventional surgeries.

## Results

Nine of the sham surgeries did not require additional surgical interventions beyond what was described in the protocol, defined in this study as “uneventful surgeries”. Three surgeries (one MVR and two AVR) required additional surgical interventions, described as “interventional surgeries”. One of the sheep in the MVR group required closure of a patent foramen ovale, one sheep in the AVR group required multiple aortotomy repairs, and the other required repair of a stab incision in the left atrial appendage and left coronary circumflex artery. The stab incision was a surgical error, uncommon in typical aortic valve replacement surgeries. Presence of a patent foramen ovale or other atrial septal defect requiring closure is a fairly common finding in the sheep model. Complicated aortotomy closure during a surgical aortic valve replacement occurs more commonly as well. As aortic sham surgeries were performed without a surgical prosthesis implanted, we would expect a larger number of complex aortic closures to be performed when a prosthesis is actually implanted, approximately 1 in 5 (20%) given a study group of 10 animals. The friable nature of the ascending aortic root and limited distance between the native aortic annulus and sinotubular junction can require multiple pledgeted repair sutures to achieve aortotomy hemostasis. All of the animals survived to the endpoint of the study at 90 days.

**Figure 1:**
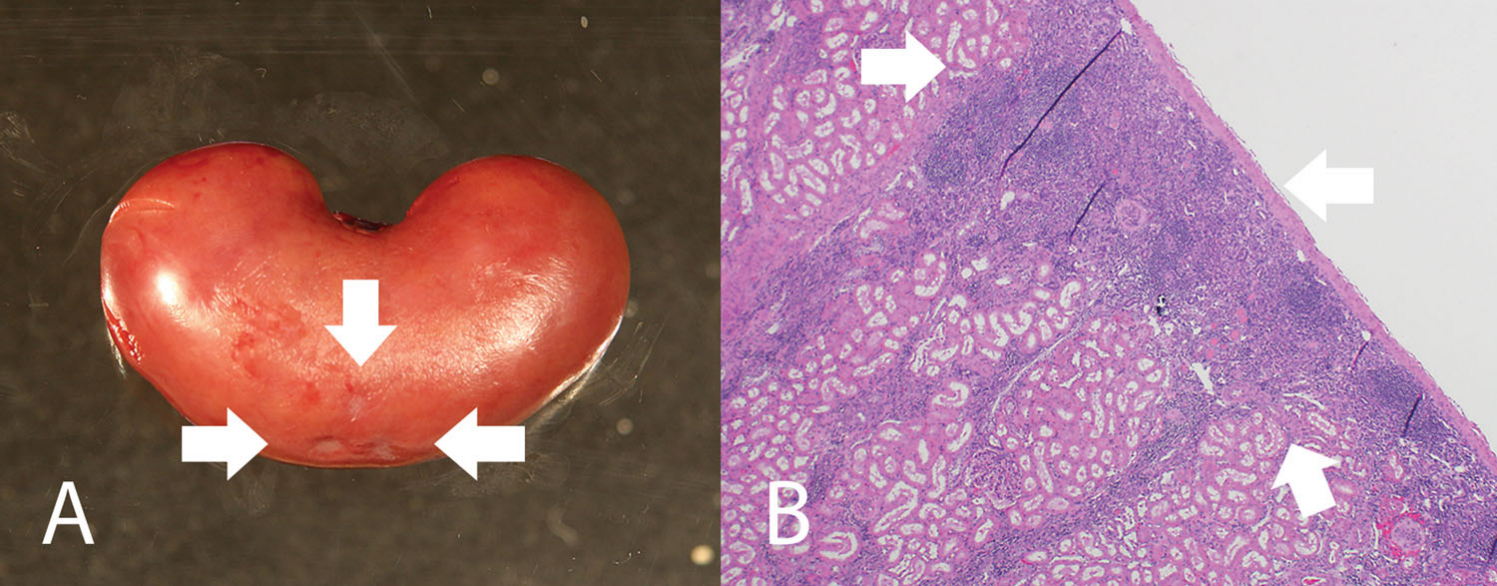
(a) Gross pathology of renal infarcts. Note well-demarcated depressed round regions of pale tan to white tissue (white arrows). (b) Histopathology of the gross lesion in (a) demonstrating wedge-shaped regions of inflammatory cell infiltration (predominantly lymphocytes with fewer plasma cells and occasional macrophages) and mild fibrosis (white arrows).

**Figure 2:**
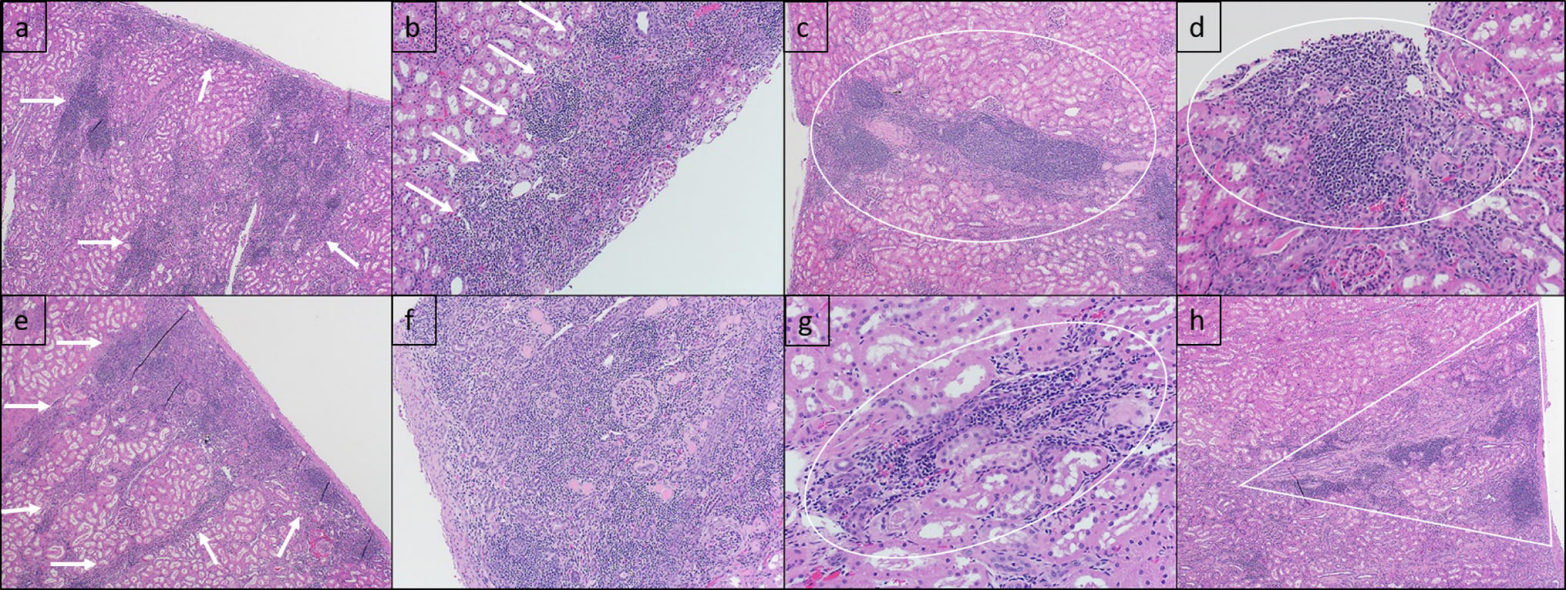
Representative histology of renal infarcts. Histology from MVR group animals 3 (a, b) and 6 (c, d) and AVR group animals 10 (e, f) and 12 (g, h). Multifocally throughout the renal cortex, renal tubules are surrounded by infiltrates of lymphocytes, plasma cells, and histiocytes and low numbers of extravasated erythrocytes (hemorrhage), as outlined by white arrows (a, b, e), white circles (c, d, g), and a white triangle in image h. The entire area represented in image f is affected.

The most commonly observed gross pathological lesion in both control and surgical sheep were small numbers of renal infarcts (RIs) as demonstrated in Fig. 1. Grossly, renal infarcts appeared as variably demarcated, flat to mildly depressed, round to irregularly round areas of pale tan to white tissue. These gross findings correlated histologically with varying degrees of cortical parenchymal loss and replacement with aggregates of lymphocytes, plasma cells, and fibrous tissue, sometimes flecked with pockets of mineralization. Select histology images are shown in Fig. 2. An underlying cause of the inflammation was not identified in any of the histopathologic samples obtained. The number of RIs correlated with total estimated area of the infarcted kidney in surgical and non-surgical control groups combined (*r* = .84, *p* < .01). All groups (non-surgical control groups, both surgical sham groups individually and combined) had a similar number of RIs (an average of 4.7 RIs in the control group, 2.5 in the MVR group, 5.8 in the AVR group, and 4.2 in MVR and AVR combined), and degree of infarcted renal area (0.95% of kidney area in controls, 0.55% in the MVR group, 0.60% in the AVR group, 0.57% in MVR & AVR combined), as shown in Table 1. The p-values from these comparisons suggest no significant difference between groups. As demonstrated in Table 2, the number of renal infarcts and percent of infarcted kidney area were significantly higher (*p* < .01) in animals requiring additional surgical interventions (average of 14.3 RIs, 1.98% infarcted area) when compared to those with uneventful surgeries (average of 0.78 infarcts, 0.10% infarcted area). Additional surgical interventions were significantly correlated with increased number of RIs (*r* = .85, *p* < .01), and area of infarcted kidney (*r* = .75, *p* < .01) when compared to uneventful surgeries. No correlation was present between number of RIs or infarcted renal area and respective type of surgery (AVR vs. MVR), time on CBP, or pre- and post-CPB blood pressure (Table 3). Renal infarction was not correlated with age or weight of the animal (Table 4). RIs were not associated with significant changes in renal function as measured by creatinine over the 90 day period (Table 5). Other commonly noted changes included mild to moderate fibrous and/or fibrinous adhesions between the left and right lungs and thoracic wall and between the pericardium and epicardium, presence of fibrous tissue and/or suture or pledget material in the left apex, right atrial appendage, and descending aorta. No gross evidence of brain lesions, or other pathologic effects of surgery, was observed.

**Table 1:** Average number and percent area of renal infarcts (RI) in each surgical group individually (MVR, AVR) and combined (MVR&AVR) compared to the control group (no surgery).

Procedure	Average number of renal infarcts	Average kidney area infarcted (%)
Control (*n* = 6)	4.7	0.95
MVR (*n* = 6)	2.5 (*p* = .50)	0.55 (*p* = .57)
AVR (*n* = 6)	5.8 (*p* = .75)	0.60 (*p* = .67)
MVR & AVR (*n* = 12)	4.2 (*p* = .96)	0.57 (*p* = .55)

Significance of each group compared to control is shown in parentheses. No significant difference in number of RIs or area infarcted were observed between the two surgical sham groups individually or combined when compared to the control group. *AVR* aortic valve replacement; *MVR* mitral valve replacement

**Table 2:** Average number of renal infarcts and percent area of infarcted kidneys in animals with uneventful surgery compared to those with surgical interventions.

Procedure	Average number of renal infarcts	Average kidney area infarcted (%)
Uneventful surgery (*n* = 9)	0.78	0.10
Surgical interventions (*n* = 3)	14.3 (*p* < .01)	1.98 (*p* < .01)

*p* values represent the comparison of animals requiring surgical interventions to those with uneventful surgeries

**Table 3:** Pearson’s correlation of renal infarcts and average kidney area infarcted respective to surgical intervention, bypass time, systolic and diastolic blood pressure.

	Surgical Intervention (*n* = 3)	Bypass Time (*n* = 12)	Pre-CPB systolic (*n* = 5)	Post-CPB systolic (*n* = 5)	Pre-CPB diastolic (*n* = 5)	Post-CPB diastolic (*n* = 5)
Average number of renal infarcts	*r* = .85	*r* = .40	*r* = − .47	*r* = .29	*r* = − .50	*r* = .26
	*p* < .01	*p* = .20	*p* = .42	*p* = .64	*p* = .40	*p* = .67
Average kidney area infarcted (%)	*r* = .75	*r* = .23	*r* = − .44	*r* = .27	*r* = − .48	*r* = .25
	*p* < .01	*p* = .47	*p* =.45	*p* = .66	*p* = .41	*p* = .68

Blood pressures were recorded on AVR model only, though one animal was excluded as it did not have blood pressure data

**Table 4:** Pearson’s correlation of RIs and respective age and weight.

	Age (months)	Weight (kg)
AVR	10.20	54.12
MVR	10.78	58.63
Control	14.12	60.97
All animals	11.70	57.91
Correlation to RIs (all animals)	*R* = 0.038	*R* = 0.035

**Table 5:** Average creatinine measured at control and 90 days (end of study), for each surgical group individually and combined.

Creatinine (mg/dL)	Control average	90 day average	Change	*p* value
MVR (*n* = 6)	0.87	0.82	− 0.5	.17
AVR (*n* = 6)	0.93	0.73	− 0.2	< 0.01
MVR & AVR (*n* = 12)	0.9	0.78	− 0.12	< 0.01

Creatinine reference interval: 0.5–1 mg/dL. *MVR* mitral valve replacement; *AVR* aortic valve replacement

A variety of other non-surgically related incidental background findings were also noted. Grossly, these included rare renal petechiae and renal cysts, small multifocal caseous filled hepatic nodules, pulmonary hypostatic congestion (dark red to purple rubbery tissue) and accessory adrenal tissue. Histologic findings included multifocal lymphoplasmacytic infiltrates within the renal interstitium, myocardium, subepidardial, subpleural, and perivascular spaces, minimal renal tubular regeneration, small pockets of mineralization within the renal medulla and pelvis, diffuse splenic red pulp expansion, diffuse alveolar collapse (atelectasis), multifocal mild biliary hyperplasia, periportal mononuclear inflammation, random small hepatic foci of inflammatory cells (polymorphonuclear cells, lymphocytes, plasma cells) with or without single cell necrosis, and mild multifocal hepatic sinusoidal congestion.

Combined AVR and MVR groups showed significant decreases in post-CPB hemoglobin (Hgb), red blood cell count (RBC), hematocrit (HCT), albumin (Alb), and globulin (Glob), as well as increases in creatinine kinase (CK) and glucose beyond the reference interval (Table 6). Both groups combined also demonstrated statistically significant changes post-CPB in creatinine, chloride, potassium, bilirubin, gamma-glutamyl transferase (GGT), cholesterol, triglycerides, beta hydroxybutyrate (BHY), lactate dehydrogenase (LDH), total white blood cell count, lymphocyte count, prothrombin time (PT) and fibrinogen (supplemental data). A list of abbreviations presented in the text is included in Table 7. All of these values were within the reference intervals of the assay and are unlikely to be of clinical relevance. No clinically pertinent changes were noted at the other time-points measured. A few sporadic statistically significant changes beyond the reference interval were seen, although the changes were very minimal and most likely to be due to normal biologic variation.

**Table 6:** Hematologic and biochemical variables showing significant post-CPB changes beyond the reference interval: RBC (red blood cells), HGB (hemoglobin), HCT (hematocrit), Creat (creatinine), Alb (albumin), Glob (globulin), CK (creatinine kinase), and Glucose.

Hematology	Reference interval	Control average	Post- CPB average	Change	*p* value
RBC (x10^6/µL)	8.95–15.25	10.1	7.74	− 2.37	< 0.01
HGB (g/dL)	9.6–14.5	9.6	7.4	− 2.2	< 0.01
HCT (%)	26.9–41.9	28.5	22.0	− 6.5	< 0.01
Biochemistry					
Alb (g/dL)	2.5–3.5	3.1	2.3	− 0.8	< 0.01
Glob (g/dL)	2.7–4.4	3.6	2.6	− 1	< 0.01
CK (U/L)	67–214	357	1204	848	< 0.01
Glucose (mg/dL)	54–103	98	109	11	0.03

**Table 7:** Abbreviations found in the text.

Abbreviation	Full form
ACT	Activated clotting time
Alb	Albumin
AVR	Aortic valve replacement
BUN	Blood urea nitrogen
CBC	Complete blood count
CK	Creatinine kinase
CPB	Cardiopulmonary bypass
GGT	Gamma-glutamyl transferase
Glob	Globulin
HCT	Hematocrit
HGB	Hemoglobin
ISO	International Organization for Standardization
LDH	Lactate dehydrogenase
MVR	Mitral valve replacement
PFO	Patent foramen ovale
PT	Prothrombin
RI	Renal infarct
SOP	Standard operating procedure

## Discussion

Sheep are the standard animal model used for the preclinical safety assessment of new or modified mitral and aortic heart valve substitutes, yet the pathologic effects of invasive surgery involving cardiopulmonary bypass (CPB) were previously undocumented. This is the first study to report expected background findings in sheep with no history of surgery and those undergoing CPB in the absence of valve replacement and establishes a baseline useful for differentiating potential device-associated changes from common procedural and background changes in sheep.

In device development, safety is often evaluated by the assessment of downstream organs, such as the brain, lungs, heart, kidneys, and liver for the presence of thromboembolic events leading to ischemic injury. Of special interest are lesions to the brain, which are a common clinical presentation of embolic events.^16^ Since no gross evidence of brain lesions were present this study, the presence of brain lesions in sheep with an implanted device would potentially be a safety concern.

In our study, the most interesting finding was the common occurrence of renal infarcts. It should be noted that the term “renal infarct” was used throughout this study as a generalized term encompassing the gross observation of variably demarcated, flat to mildly depressed, round to irregularly round areas of pale tan to white tissue that histopathologically correlated with the finding of tubulointerstitial mononuclear (lymphocytes, plasma cells, histiocytes) inflammation and varying degrees of interstitial fibrosis, tubular atrophy, and mineralization.

The finding of renal infarcts is important in device development, as the degree of infarction is often used a potential indicator of downstream thromboembolic complications associated with the presence of the device. In the authors’ experience, renal infarcts are often noted at sacrifice in study animals without a definitive underlying cause. In humans, renal infarcts after cardiac surgery are suspected to result from a combination of decreased renal oxygenation and perfusion, altered hemodynamics, and embolic events occurring from gas bubbles, biologic aggregates, inorganic debris, microthromboemboli, and/or manipulation of the aorta during cannulation.^5^,^8^,^14^ We hypothesized that a similar process may be occurring in sheep, and wanted to investigate the degree of renal infarction that could be expected due to surgery with CPB versus control. Interestingly, renal infarction was similar between both the nonsurgical control and surgical MVR and AVR groups. This finding suggests that the majority of renal infarcts in sheep are not actually related to the surgical procedure and are present from other non-surgical causes. This is supported by the finding that the number of renal infarcts were not associated with type of surgical procedure (MVR vs AVR), time on CPB, or pre- and post-surgical blood pressures (hypoperfusion).

We did find that sheep with additional surgical interventions had significantly higher numbers of RIs compared to the other groups. This suggests that additional surgical handling and manipulation of the heart and associated vasculature is associated with a greater number of lesions and should be taken into consideration when interpreting study results regarding device safety. It is also possible that more complex procedures may lead to greater numbers of RIs. In our study we manipulated the native valve leaflets but did not undergo removing the leaflets and replacing them with a replacement valve, but in testing situations this would be performed. In device studies, these added interventions may lead to an increased number of RIs, but this would need further validation.

An underlying cause of the renal infarcts was not found histologically and unfortunately, a definitive etiology could not be determined in this study. Reported causes of renal infarcts in sheep are vague and general, including any cause of embolic or thrombotic occlusion to the renal arteries.^11^,^13^,^15^,^19^ The only published report in sheep of a non-surgical related cause of renal infarction was due to infection with *Mycoplasma mycoides* subsp. mycoides, a causative agent of respiratory disease.^20^ Several reports in the literature refer to the common occurrence of renal infarcts in sheep, particularly identified at slaughter, however these studies did not perform further analysis or speculation regarding causes of the renal infarcts beyond the potential of septic emboli from previous bacterial infections.^2^,^7^ The lack of a definitive cause of infarcts in sheep is likely a combination of the abundance of underlying causes and difficulty in determining when the infarction occurred. Causes of thrombosis in all species include any factor altering the components of Virchow’s triad (vascular injury, hypercoagulability, and abnormal blood flow), with endothelial damage being cited as the most important factor.^13^,^15^ Potential contributors to endothelial damage are vast, including trauma, vasculitis, metabolic disorders, neoplasms, and toxins.^15^ The only specific, reported causes in cattle include necrotizing toxins released by pathogenic bacteria (*Manheimia haemolyticia*) and ergot poisoning.^15^ When considering the general causes of infarcts, along with reported causes in cattle, it could be hypothesized that a variety of commonly encountered diseases in sheep could result in renal infarcts. Of highest consideration would be bacterial organisms resulting in numerous pathologies including Johne’s disease (*Mycobacterium avium* subspecies *paratuberculosis), enterotoxemia (Clostridium perfringens), respiratory disease (Mannheimia haemolytica*, *Pasteurella multocida), caseous lymphadenitis (Corynebacterium ovis), and foot rot (*Dichelobacter nodosus and Fusobacterium necrophorum). Other considerations include, but are not limited to, improper diet (rumen acidosis), viral disease (caprine arthritis encephalitis), parasites (Haemonchus contortus, coccidiosis), and fungal disease (fescue toxicosis).^17^,^21^ In addition to the myriad of potential underlying causes, identifying an underlying etiology to renal infarcts is further complicated by the difficulty in narrowing down the timeframe in which the infarct occurred. The infarcted tissue rapidly transforms from red in the acute phase, to a complete white discoloration as early as two-three days after the initial ischemic event, thus precluding the ability to differentiate between infarcts caused by recent events from those that may have occurred prior to acquisition of the animal for the study.^13^ Furthermore, histology can rarely be useful in differentiation of the causes of renal infarcts, as infarcted tissue can appear identical to focal healed pyelonephritis and the inability to assess every renal artery for the presence of thromboemboli.^13^

Although an underlying cause of the RIs was not determined, the main purpose of this study was to establish the typical number and degree of renal infarcts unrelated to the presence of a device in sheep. Additional studies would be required to further investigate the potential causes of renal infarcts in experimental sheep. Importantly, RIs were not associated with a significant increase in creatinine over 90 days in any of the animals. In fact, the creatinine of all animals significantly decreased over the course of the study, indicating that deconditioning and loss of muscle mass in the animals after surgery had a greater impact on serum creatinine than the presence of RIs. Therefore, the overall impact of the RIs regardless of underlying cause can be considered mild.

In addition to gross and histopathologic changes, we identified numerous deviations in hematology and serum biochemical parameters associated with surgery. Post-CPB, both groups showed statistically significant decreases in hemoglobin (HGB), red blood cell count (RBC), hematocrit (HCT), albumin (Alb), and globulin (Glob), and increases in creatine kinase (CK) and glucose beyond the reference interval. Decreases in HGB, RBC, HCT, Alb, and Glob are expected due to a combination of blood loss during surgery and dilutional effects from CPB. An increase in CK is indicative of muscle damage, and is a common finding in sheep after handling, transport, injections, prolonged recumbency, and surgical trauma. It should be noted that an increase of 5–6 × the upper end of the reference interval was seen due to surgical intervention. These background increases are important to take into consideration if using CK as a marker of myocardial infarction. The increased glucose is likely attributable to increased stress post-operatively. A few sporadic statistically significant changes beyond the reference interval were seen, but the changes were very minimal and most likely to be due to normal biologic variation. Both groups demonstrated statistically significant changes in values at many of the time-points measured, but these remained within the reference interval and are less likely to have clinical relevance. Similarly, there was a statistically significant difference in the change of several values between the AVR and MVR groups, although the majority were still within the reference range. Those that extended beyond the reference range were minimal and were not clinically relevant.

One limitation to this study is the small number of animals used. We attempted to use a large enough number of animals so that statistically significant comparisons could be performed, while operating within the financial constraints of the study budget. Best practices for the use of animals in biomedical research recommend that a minimum number of animals be used in a study to achieve statistical results. A second limitation of the study is that only one breed of domestic sheep was used in the study, the Columbia Cross breed. This is a breed of domestic sheep commonly used in research, but there are a wide variety of other domestic sheep breeds used as chronic animal models. The assumption that the Columbia Cross breed is no different than other domestic sheep breeds in terms of baseline pathophysiologic observations is a limitation to the current study. Finally, three animals in this study underwent additional surgical interventions than what was planned in the study protocol. Two of these interventions (PFO and complicated aortotomy closure) are two common occurrences encountered in valve replacement surgery in the sheep model. Had a valve prostheses been implanted during aortic surgeries, we would expect a greater incidence of complicated aortotomy closures than what was observed in the present study. While there was a significant correlation between additional surgical interventions and number of renal infarcts, the small number of animals requiring additional surgical intervention may underestimate the significance of this finding.

An additional limitation was the lack of exact measurements of each of the kidneys, primarily the height, in order to determine a more accurate estimation of the area of each kidney infarcted. However, the normal “kidney bean” shape, along with normal biologic variation between different areas of the kidney (for example the cranial pole is often smaller than the caudal pole), precludes the determination of an exact renal volume, thus requiring the use of an estimation regardless. Although a limitation, an exact determination of affected area was not deemed to be critical to the interpretation of the results of this study and was used primarily to validate that the number of renal infarcts correlated with the area of kidney infarcted (in comparison to a small number of infarcts affecting a large area of the kidney). Furthermore, a more thorough examination for any current or previous condition that may contributed to the formation of renal infarcts would have been valuable but given the extent of potential underlying causes was beyond the scope of this study.

In this study, we established a set of expected baseline background changes of both nonsurgical control sheep and sheep undergoing a sham MVR and AVR surgery. This data can be used as a guideline for safety evaluation in development of heart valve substitutes in both industry and academic institutions. Importantly, we discovered that a degree of renal infarction can be expected in sheep, and that additional surgical interventions not directly related to the presence of the device can increase the number of RIs. Given the importance of identifying device-related thromboembolic events for safety assessment, it is critical to take into consideration the expected amount of renal infarction from background etiologies in determining those that are potentially a device-associated safety concern. Furthermore, we have indicated the expected blood chemistry and hematology values for surgery, which can be compared to values from implanted devices that may be associated with systemic pathology such as biologic immunoreactivity with tissue engineered valves, systemic inflammation, or hemolysis.

## Conclusion

This study shows the background pathologic effects in control sheep and those undergoing cardiopulmonary bypass and open cardiac surgery. We determined a baseline level of RIs expected to be seen in sheep to aid in determining the presence of device-related thromboembolic events during preclinical safety assessment. We also identified that complex surgical interventions may lead to an increased average number of renal infarcts, but RIs are not associated with mitral vs aortic valve replacement, time on CPB or blood pressure. Despite the common occurrence of background RIs, the overall effects are mild and are not associated with changes in blood values used to assess renal function. Additionally, expected deviations in blood values outside of the reference interval can be expected post-CPB in hemoglobin, red blood cell count, hematocrit, albumin, globulin, creatine kinase, and glucose. This data establishes a critical set of guidelines useful for determining preclinical safety concerns in the development of novel heart valve replacement devices in the sheep model.

## Supplementary Information

Below is the link to the electronic supplementary material.

Supplementary material 1 (DOCX 15 kb)
