# Supplementary material for: Establishing Background Pathologic Changes of Valve Replacement Surgery in Sheep
Source: Cardiovasc Eng Technol. 2021 Jul 14;13(1):181–90. doi: 10.1007/s13239-021-00563-6 (PMC8888364; doi:10.1007/s13239-021-00563-6)
Supplement: Supplementary file 1 — Supplementary material 1 (DOCX 15 kb) [file 13239_2021_563_MOESM1_ESM.docx]

| Analyte | Reference Range | Control Mean  (n=12) | Post-Bypass Mean  (n=12) | Change | p-value |
| --- | --- | --- | --- | --- | --- |
| Creat (mg/dL) | .5-1 | 0.9 | 1.04 | 0.14 | <0.01 |
| Cl (mmol/L) | 100-110 | 105.0 | 107.8 | 2.8 | 0.0054 |
| K (mmol/L) | 4-5.4 | 4.0 | 4.5 | 0.5 | 0.0023 |
| T. Bili (mg/dL) | .1-.2 | 0.19 | 0.10 | -0.09 | <.0001 |
| GGT (U/L) | 39-110 | 61.9 | 45.7 | -16.3 | <.0001 |
| Chol (mg/dL) | 34-95 | 59.9 | 46.5 | -13.4 | 0.0149 |
| Trig (mg/dL) | 11-40 | 128.3 | 35.1 | -93.3 | <.0001 |
| BHY (mmol/L) | <.67 | 0.41 | 0.33 | -0.08 | 0.0479 |
| LDH (U/L) | 348-680 | 464.7 | 419.9 | -44.8 | 0.0444 |
| WBC (x10^3^/µL) | 4.2-13 | 7.1 | 5.5 | -1.6 | 0.0005 |
| #LYMPH (x10^3^ cells/µL) | 1.4-6.3 | 4.3 | 2.7 | -1.6 | <.0001 |
| PT (sec) | NA | 11.8 | 12.2 | 0.4 | 0.044 |
| Fibrinogen (mg/dL) | 104-390 | 241.0 | 181.1 | -59.9 | 0.0037 |

Supplemental Table 1: Hematologic and biochemical variables showing significant post-CPB changes falling within the reference interval in both sham surgical groups combined: Creat (creatinine), Cl (chloride), K (potassium), T.Bili (total bilirubin), GGT (gamma-glutamyl transferase), Chol (cholesterol), Trig (triglycerides), BHY (beta hydroxybutyrate), LDH (lactate dehydrogenase), WBC (white blood cells), LYMPH (lymphocytes), PT (prothrombin time).
